# Mercury in the human thyroid gland: Potential implications for thyroid cancer, autoimmune thyroiditis, and hypothyroidism

**DOI:** 10.1371/journal.pone.0246748

**Published:** 2021-02-09

**Authors:** Roger Pamphlett, Philip A. Doble, David P. Bishop

**Affiliations:** 1 Discipline of Pathology, Sydney Medical School, Brain and Mind Centre, The University of Sydney, Sydney, New South Wales, Australia; 2 Department of Neuropathology, Royal Prince Alfred Hospital, Sydney, New South Wales, Australia; 3 Elemental Bio-Imaging Facility, School of Mathematical and Physical Sciences, University of Technology Sydney, Sydney, New South Wales, Australia; University of Louisville School of Medicine, UNITED STATES

## Abstract

**Objective:**

Mercury and other toxic metals have been suggested to be involved in thyroid disorders, but the distribution and prevalence of mercury in the human thyroid gland is not known. We therefore used two elemental bio-imaging techniques to look at the distribution of mercury and other toxic metals in the thyroid glands of people over a wide range of ages.

**Materials and methods:**

Formalin-fixed paraffin-embedded thyroid tissue blocks were obtained from 115 people aged 1–104 years old, with varied clinicopathological conditions, who had thyroid samples removed during forensic/coronial autopsies. Seven-micron sections from these tissue blocks were used to detect intracellular inorganic mercury using autometallography. The presence of mercury was confirmed using laser ablation-inductively coupled plasma-mass spectrometry which can detect multiple elements.

**Results:**

Mercury was found on autometallography in the thyroid follicular cells of 4% of people aged 1–29 years, 9% aged 30–59 years, and 38% aged 60–104 years. Laser ablation-inductively coupled plasma-mass spectrometry confirmed the presence of mercury in samples staining with autometallography, and detected cadmium, lead, iron, nickel and silver in selected samples.

**Conclusions:**

The proportion of people with mercury in their thyroid follicular cells increases with age, until it is present in over one-third of people aged 60 years and over. Other toxic metals in thyroid cells could enhance mercury toxicity. Mercury can trigger genotoxicity, autoimmune reactions, and oxidative damage, which raises the possibility that mercury could play a role in the pathogenesis of thyroid cancers, autoimmune thyroiditis, and hypothyroidism.

## Introduction

Environmental factors are estimated to contribute more that 40% to the risk of thyroid cancers [1] and about 25% to the risk of autoimmune thyroiditis [2]. The nature of these risk factors remains largely unknown [3], but one topic of interest has been the possibility that heavy metals may play a role in thyroid disorders since the thyroid gland appears to be a target for environmental toxic metals [4, 5]. Evidence for a role for toxic metals comes from epidemiological and experimental studies showing the effects of toxic metals on thyroid function, with most studies focusing on mercury, cadmium, and lead [4, 6–8]. However, conflicting results, underpowered epidemiological studies, problems in establishing environmental sources of heavy metal exposure, and difficulties in translating the findings of animal toxicant studies to human toxicant exposure, have hampered research in this field. Animal studies indicate that the thyroid gland of the rat, dog and monkey are predisposed to take up heavy metals such as mercury [5, 9, 10], but animal studies cannot replicate the human experience of being exposed long-term to continuous or repeated low levels of toxic metals that bioaccumulate in tissues. They also do not account for potential human genetic susceptibilities to heavy metal toxicity [11, 12]. Finding environmental influences on thyroid disorders remains an important topic, particularly since many of these disorders are reported to be increasing in incidence [1, 13–17], raising suspicion that environmental pollutants may contribute to this increase [3].

Few studies of toxic metals in the human thyroid are available [5, 18, 19]. Levels of mercury in autopsy-sampled thyroid, measured by atomic absorption, were five times higher in people with more than 11 occlusal mercury-containing amalgam fillings than people with fewer than four such fillings [18]. Similarly, levels of mercury in autopsy-sampled thyroids, measured by inductively coupled plasma-mass spectrometry, correlated with the numbers of dental amalgam surfaces present [19]. In tissue samples removed at surgery from euthyroid subjects, levels of elements such as mercury and cadmium, measured with inductively coupled plasma-mass spectrometry, were higher in the thyroid than in adjacent muscle and fat samples [5]. In these studies, thyroid tissue had to be digested before analysis so elements could not be located within specific cells. This is relevant to the thyroid, since its follicular architecture means the overall cellularity of the thyroid is low, so elements present at low levels, or at high levels in only a few cells, are difficult to detect.

To gain a clearer picture of the role of toxic metals in thyroid disorders, we designed a project with two differences to previous studies. First, we used two elemental bio-imaging techniques that allowed us to study the cellular distribution of mercury and other toxic metals in sections of human thyroid glands. Second, we analysed autopsy-derived thyroid samples from people with a spectrum of clinicopathological conditions, and from a wide range of ages, from which we could estimate the prevalence of mercury-containing thyroid glands and the effect of aging on the mercury content of the thyroid.

## Materials and methods

### Ethics

This study (X14-029) was approved by the Human Research Committee, Sydney Local Health District (Royal Prince Alfred Hospital Zone). This institutional review board waived the need for written informed consent from relatives of individuals studied since this was a de-identified retrospective study of archived paraffin-embedded tissue. Data were fully anonymised on the research database after initial access to Department of Forensic Medicine records.

### Sample collection

Paraffin-embedded thyroid tissue blocks were obtained from The New South Wales Department of Forensic Medicine tissue archive. These had been taken as part of standard tissue sampling from the autopsies of 115 people (68 male, 47 female) with a mean age of 54 years, median age of 47 years, age range 1–104 years, and SD 27 years (**Table 1**). Major medical conditions were: none known (N = 50), neurodegenerative disease (N = 33), psychosis (N = 27), epilepsy (N = 2), and one each of anorexia nervosa, cancer, and Down syndrome. Causes of death were: suicide (N = 26), trauma (N = 17), drowning (N = 16), cardiovascular (N = 14), drug overdose (N = 14), infection (N = 8), undetermined (N = 6), choking (N = 5), cerebrovascular (N = 3), cancer (N = 2), and one each of hypothermia, respiratory insufficiency, sudden unexpected death from epilepsy, and undernutrition.

**Table 1:** Age, gender, and follicular cell autometallography of samples.

ID	Age	Gender	AMG	ID	Age	Gender	AMG	ID	Age	Gender	AMG
T1	1	Female	0	T40	39	Male	++	T79	70	Male	+
T2	2	Male	0	T41	39	Male	0	T80	71	Female	0
T3	4	Male	0	T42	39	Male	0	T81	72	Female	++
T4	9	Male	0	T43	40	Female	0	T82	72	Female	0
T5	16	Male	0	T44	40	Female	0	T83	74	Male	+
T6	18	Male	0	T45	41	Male	0	T84	74	Female	++
T7	18	Female	0	T46	41	Female	0	T85	75	Male	0
T8	18	Female	0	T47	41	Male	0	T86	76	Female	++
T9	20	Male	0	T48	42	Male	0	T87	77	Female	+
T10	20	Male	0	T49	43	Male	0	T88	77	Female	0
T11	20	Female	0	T50	43	Male	0	T89	77	Male	0
T12	20	Male	0	T51	43	Male	0	T90	80	Male	0
T13	21	Female	0	T52	44	Male	0	T91	81	Female	++
T14	22	Female	0	T53	44	Female	0	T92	83	Male	++
T15	23	Male	0	T54	45	Male	++	T93	86	Male	+
T16	24	Male	0	T55	45	Male	0	T94	86	Female	0
T17	25	Female	0	T56	45	Male	0	T95	87	Female	0
T18	26	Female	0	T57	46	Female	0	T96	89	Female	+
T19	26	Male	0	T58	47	Male	0	T97	95	Female	0
T20	28	Male	0	T59	47	Male	+	T98	95	Female	0
T21	29	Female	0	T60	48	Female	0	T99	95	Male	0
T22	29	Male	0	T61	49	Female	+	T100	95	Female	++
T23	29	Male	++	T62	49	Male	0	T101	95	Female	+
T24	30	Male	0	T63	49	Male	0	T102	96	Female	0
T25	30	Male	0	T64	49	Male	0	T103	96	Male	+
T26	30	Male	0	T65	53	Male	0	T104	96	Male	0
T27	33	Male	0	T66	54	Male	0	T105	96	Female	0
T28	34	Male	0	T67	55	Male	0	T106	96	Female	0
T29	35	Male	0	T68	58	Male	0	T107	97	Female	0
T30	35	Female	0	T69	59	Female	0	T108	97	Female	0
T31	35	Female	0	T70	59	Male	0	T109	97	Female	0
T32	36	Male	0	T71	61	Male	0	T110	98	Male	0
T33	36	Female	0	T72	61	Male	0	T111	98	Male	+
T34	37	Male	0	T73	61	Female	++	T112	99	Male	0
T35	37	Male	0	T74	62	Male	++	T113	100	Male	0
T36	37	Female	0	T75	66	Male	0	T114	104	Female	0
T37	38	Female	0	T76	67	Male	+	T115	104	Female	0
T38	38	Male	0	T77	69	Male	0				
T39	38	Female	0	T78	70	Male	0				

Age: years, AMG: autometallography staining of follicular cells, ID: sample identity number, 0: no AMG-positive follicular cells, +: AMG-positive follicular cells but fewer than five follicles with 50% or more AMG-positive cells, ++: at least five follicles with 50% or more AMG-positive cells.

### Autometallography

Paraffin blocks were sectioned at 7 μm with a Feather S35 stainless steel disposable microtome blade and deparaffinised. Sections were stained for inorganic mercury using silver nitrate autometallography, which represents the presence of mercury as black silver grains surrounding the mercury [20]. Autometallography is a sensitive amplification technique that can detect as few as 10 mercury sulphide/selenide molecules in a cell [21]. Briefly, sections were placed in physical developer containing 50% gum arabic, citrate buffer, hydroquinone and silver nitrate at 26°C for 80 min in the dark then washed in 5% sodium thiosulphate to remove unbound silver. Sections were counterstained with mercury-free hematoxylin and viewed with bright-field microscopy. Each staining run included a control section of mouse spinal cord where motor neuron cell bodies contained mercury following an intraperitoneal injection of mercuric chloride, with archived paraffin blocks used from a previous experiment approved by the University of Sydney Animal Ethics Committee [22]. Sections were stained with hematoxylin only to act as a control for the autometallography-stained sections, to ensure any black grains seen were from the autometallography and not from the melanin-like pigment that can occasionally be seen in follicular cells [23]. Autometallography staining of the thyroid was categorised as either: 0: no AMG-positive follicular cells, +: AMG-positive follicular cells but fewer than five follicles with 50% or more AMG-positive cells, or ++: at least five follicles with 50% or more AMG-positive cells.

### Laser ablation-inductively coupled plasma-mass spectrometry (LA-ICP-MS)

To confirm which metal autometallography was demonstrating (since autometallography can also detect inorganic silver and bismuth) and to look for the presence of other toxic metals, 7 μm paraffin sections of selected thyroid samples were deparaffinised and subjected to LA-ICP-MS for mercury, silver, bismuth, aluminium, gold, cadmium, chromium, iron, nickel and lead, as well as for phosphorus (contained in cell nuclei) to assess cellular density. Analyses were carried out on a New Wave Research NWR-193 laser or a Teledyne Cetac LSX-213 G2+ laser hyphenated to an Agilent Technologies 7700x ICP-MS, with argon used as the carrier gas. LA-ICP-MS conditions were optimised on NIST 612 Trace Element in Glass CRM and the sample was ablated with a 50 μm spot size and a scan speed of 100 μm/s at a frequency of 20 Hz. The data were collated into a single image file using in-house developed software and visualised using FIJI.

### Statistical analyses

Prism v8.4 software was used for chi-square analyses with Fisher’s exact test to compare categorical variables, chi-square analysis for trend to look for age-effects in groups, and t-tests to compare continuous variables. Significance was assessed at the 0.05 level.

## Results

### Autometallography

**Fig 1:**
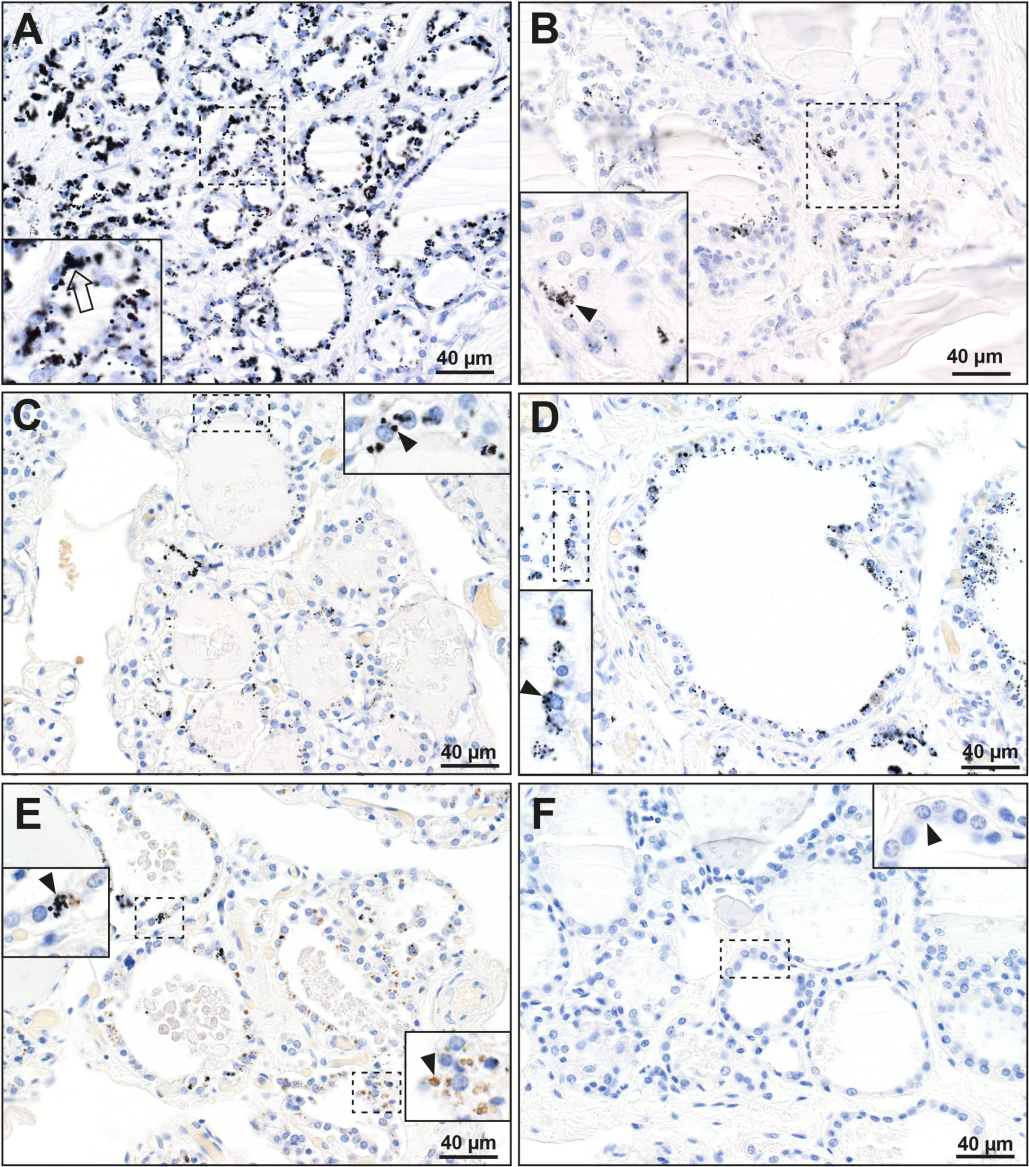
Mercury staining in the thyroid gland. Insets are enlarged views from the dashed-line rectangles. (**A**) Dense mercury grains (eg, arrow in inset) are present in most cells of these thyroid follicles (T81). (**B**) Another region of the thyroid in sample A shows mercury grains in only a few scattered follicular cells (eg, arrowhead), showing the variability of mercury uptake in different regions of the thyroid (T81). (**C)** Black mercury grains (eg, arrow) are seen in fewer that 50% of the follicular cells in this thyroid (T87). (**D**) Multiple small mercury grains (eg, arrowhead) are present in most cells of this follicle (T23). (**E**) In some follicles, a mixture of cells containing mercury (arrowhead, left) and lipofuscin (eg, arrowhead, right) were seen (T103). (**F**) No black mercury grains are seen in the cells (eg, arrowhead) of these follicles (T97). Autometallography/hematoxylin. T: sample identity number (see Table 1).

Mercury-staining black grains were seen in the cytoplasm of follicular cells in 22 of the 115 (19%) samples, 15 with category + and 7 with category ++ autometallography (the latter all aged over 70 years) (**Fig 1, Table 1**). The density of mercury staining in individual follicular cells varied within and between samples. The proportion of follicular cells containing mercury also varied within samples, as well as between samples.

### Proportion of people with mercury in their thyroid glands

**Fig 2:**
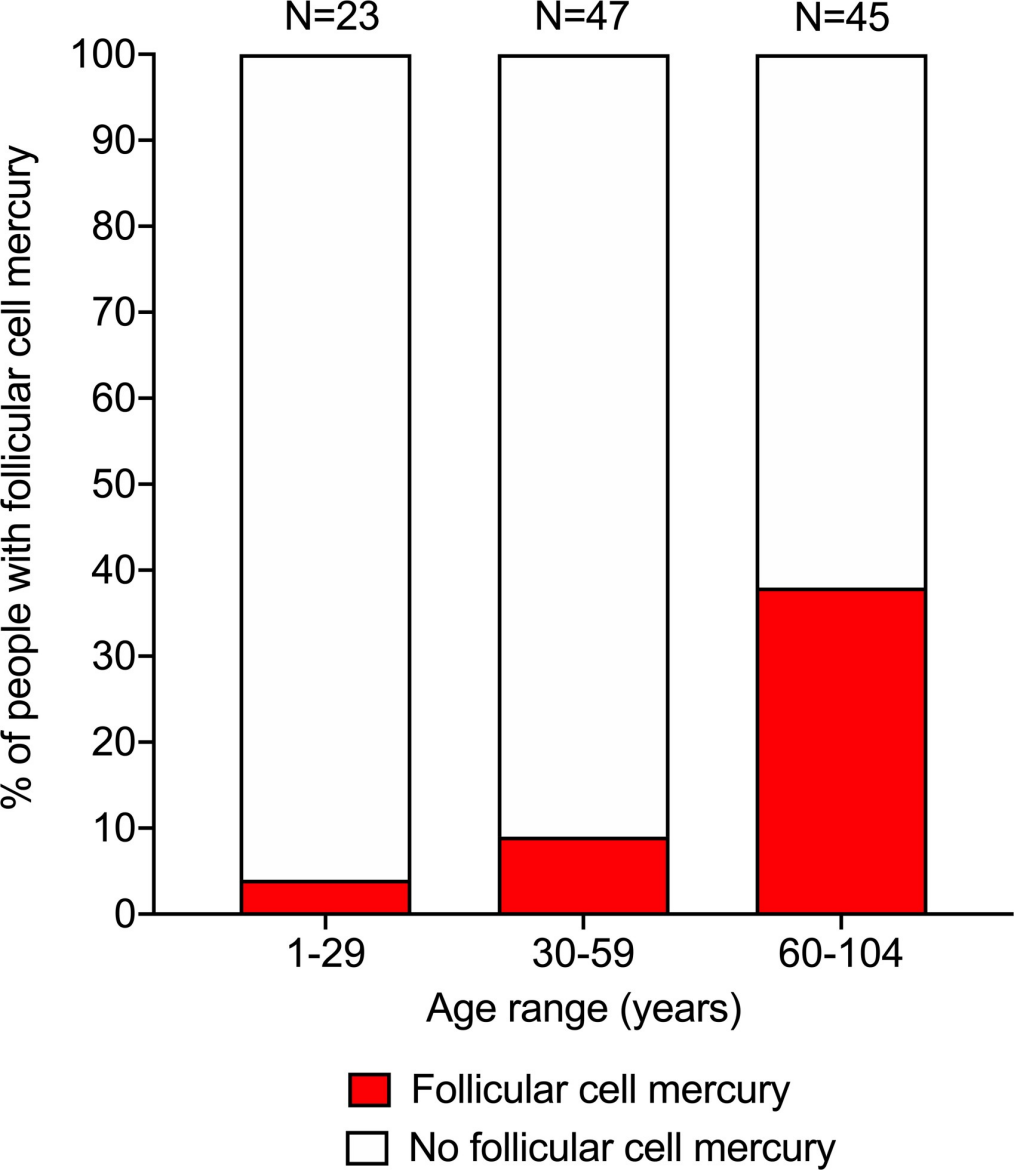
Proportion of people with follicular cells containing mercury. Mercury was seen on autometallography in the thyroid follicular cells of 4% of people aged between 1–29 years old, 9% of people aged between 30–59 years old, and 38% of people aged between 60–104 years old. Numbers above bars = numbers in age groups.

The proportion of people with mercury in their thyroid follicular cells was 4% in the 1–29 years age range, 9% in the 30–59 years age range, and 38% in the 60–104 years age range (trend p <0.0001) (**Fig 2**). The mean age of people with thyroid follicular cell mercury was higher (mean age 71 years, SD 20 years, range 29 to 98 years) than those without mercury (mean age 50 years, SD 28 years, range 1–104 years) (p = 0.001). The proportion of thyroid samples containing mercury did not differ between males (12 out of 68, 18%) and females (10 out of 37, 21%) (p = 0.64), despite females having a higher mean age (61 years, SD 30 years) than males (49 years, SD 25 years) (p = 0.028). There were insufficient numbers in subgroups of pre-mortem medical conditions, or of causes of death, to undertake robust statistical analysis of thyroid mercury in these subgroups.

### LA-ICP-MS

**Fig 3:**
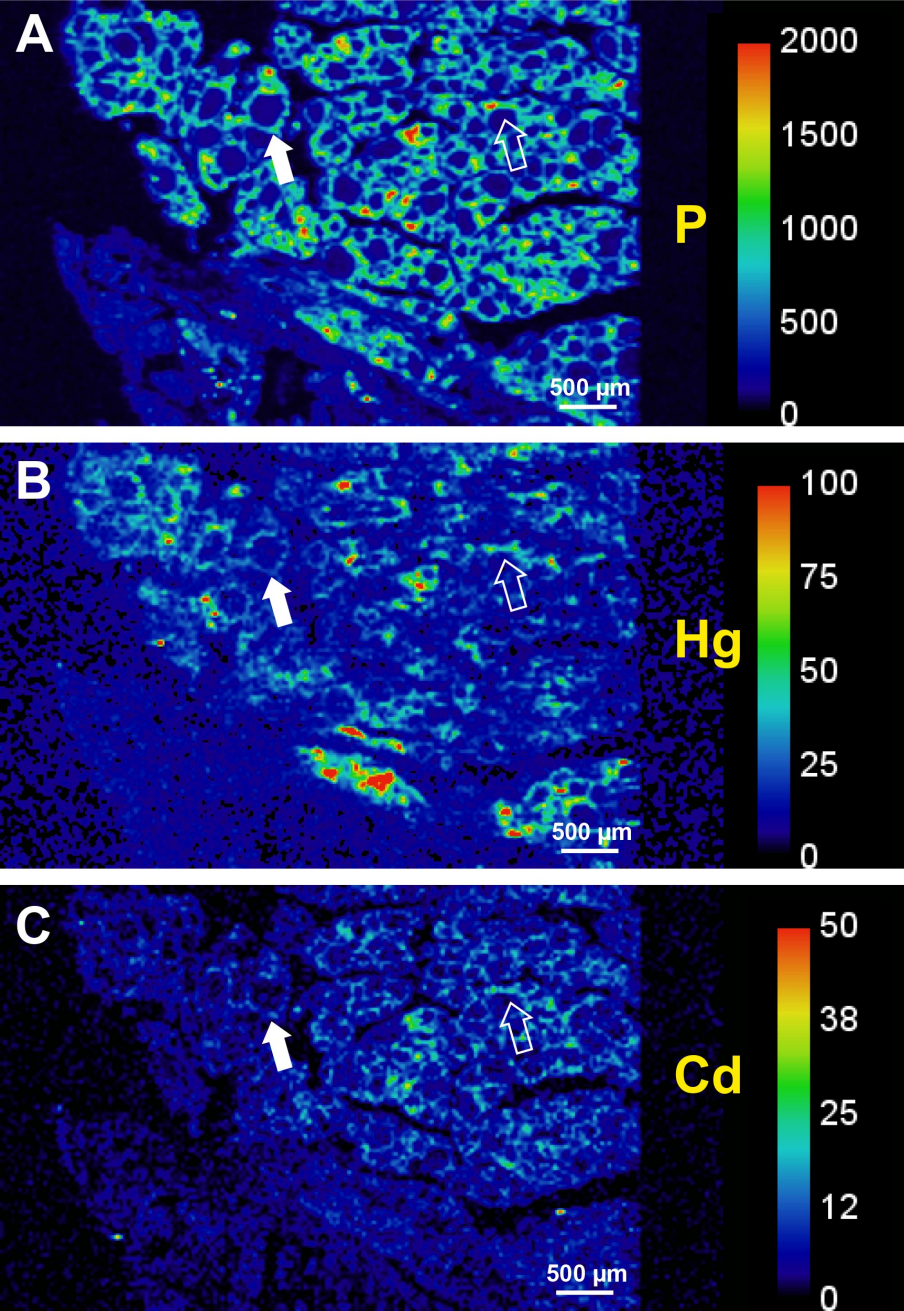
Localisation of mercury and cadmium with LA-ICP-MS. This sample (T40) showed autometallography staining of most thyroid follicles. (**A**) Phosphorus imaging of cell nuclei demonstrates the follicular architecture of the thyroid. The filled arrow shows an example of one complete follicle. The open arrow shows cells at one edge of a follicle. (**B**) Mercury is present in most follicular cells. (**C**) Cadmium is present in scattered follicular cells. Scale = counts per second (proportional to abundance). T: sample identity number (see Table 1).

**Fig 4:**
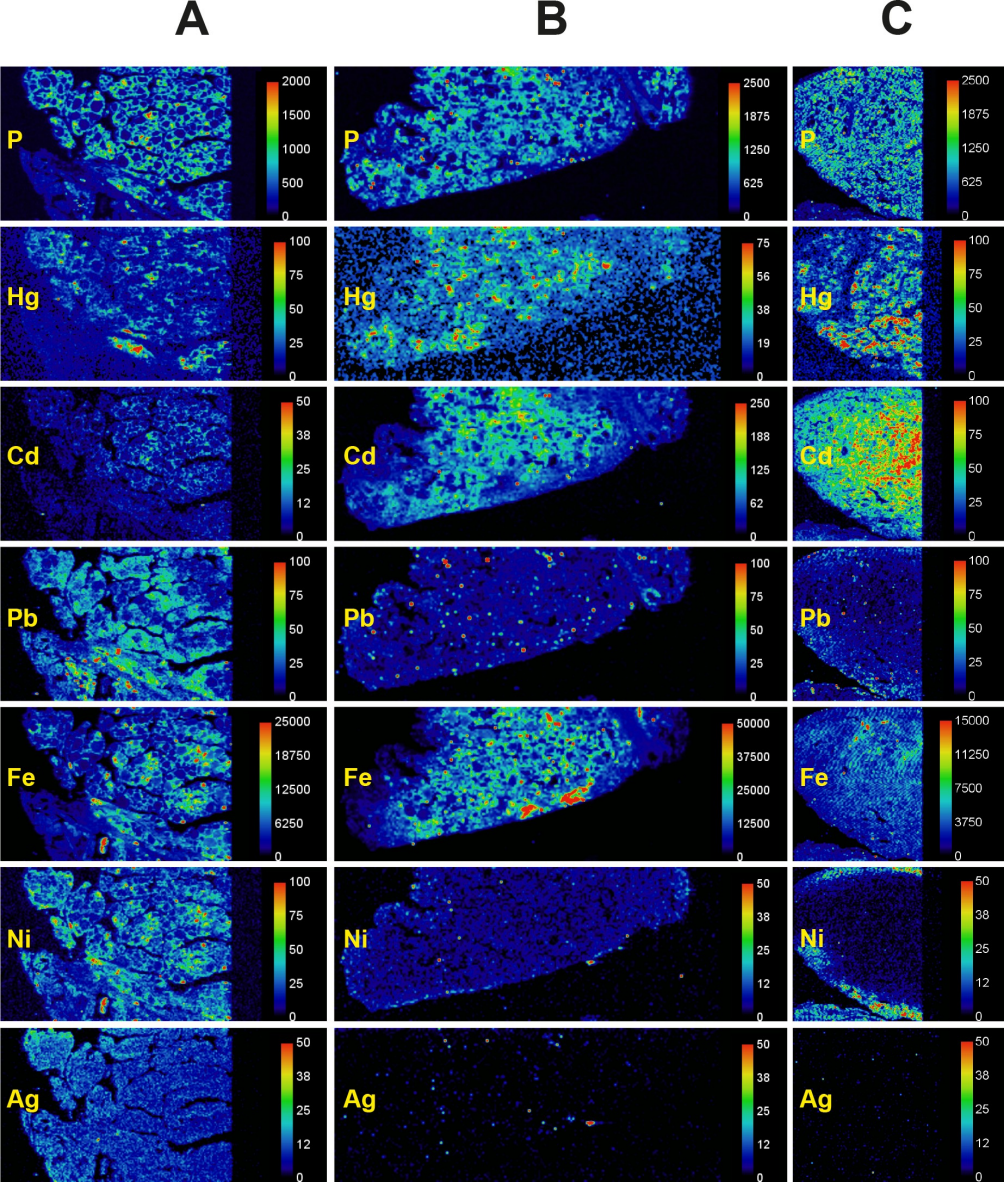
LA-ICP-MS of AMG-positive thyroid samples. Phosphorus images show the cellularity of the samples. **(A)** Follicular cells contain mercury, cadmium, lead, iron, and nickel (T40). **(B)** Follicular cells contain mercury, cadmium and iron (T86). **(C)** Follicular cells contain mercury, cadmium and iron (T91). Small discrete red dots, eg, in the lead image in B, are from surface contamination. An artefactual nickel edge effect is seen in C. Scale = counts per second (proportional to abundance). T: sample identity number (see Table 1).

**Fig 5:**
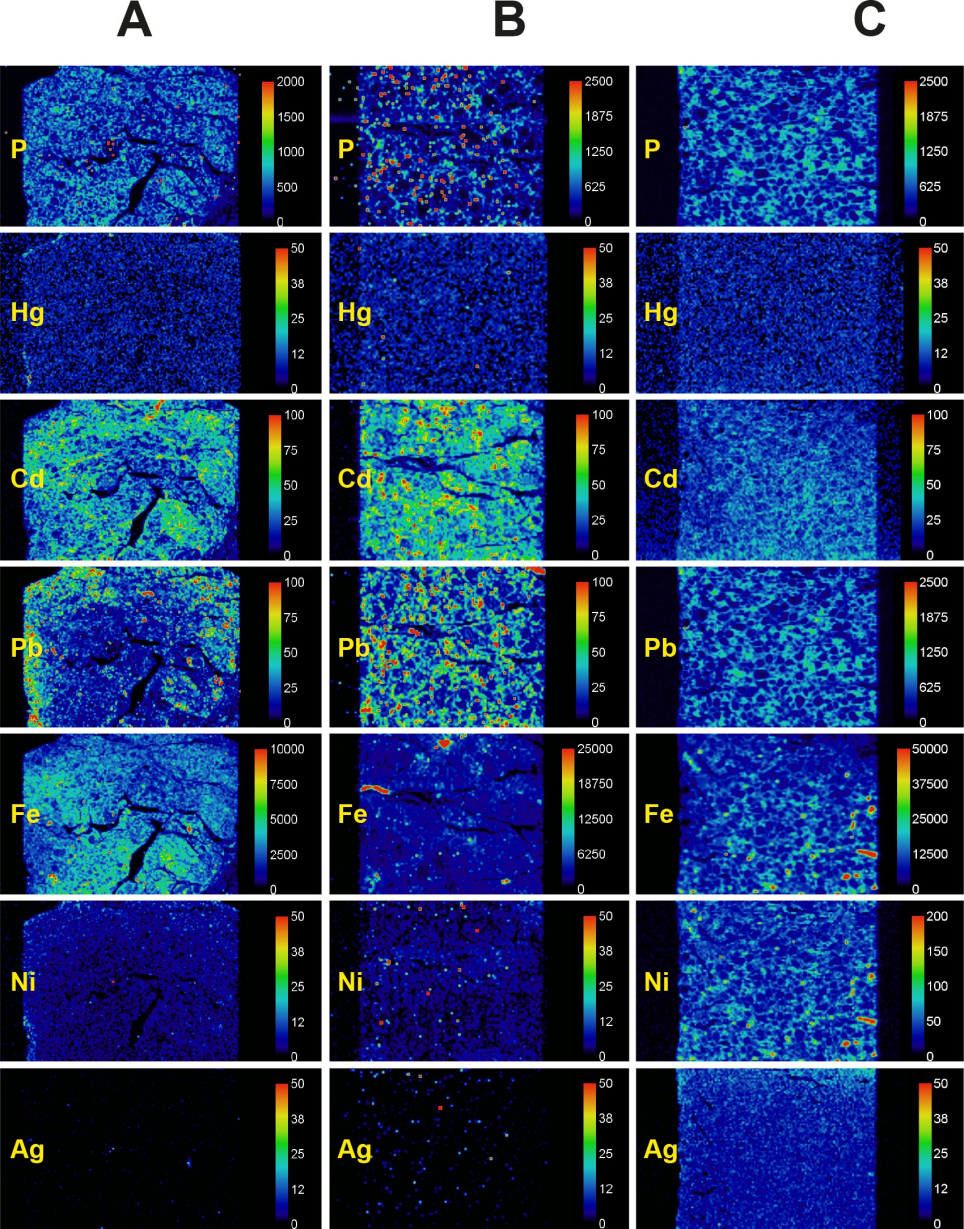
LA-ICP-MS of AMG-negative thyroid samples. Phosphorus images show the cellularity of the samples. **(A)** Cadmium lead and iron are present in follicular cells (T97). **(B)** Cadmium, lead and iron (sporadically) are present in follicular cells (T45). **(C)** Follicular cells contain cadmium, lead, iron and nickel (T70). Scale = counts per second (proportional to abundance). T: sample identity number (see Table 1).

LA-ICP-MS phosphorus images demonstrate cell nuclei and outlined the follicular architecture of the thyroid (**Fig 3**). Metals such as mercury and cadmium could therefore be localised to follicular cells using LA-ICP-MS (**Fig 3**). LA-ICP-MS images showed follicular cell mercury in all the three autometallography-positive thyroid samples (**Fig 4, Table 2**), but no LA-ICP-MS mercury was seen in three samples that did not stain with autometallography (**Fig 5, Table 2**). Apart from mercury, four other potentially toxic metals were detected in the six LA-ICP-MS samples (**Figs 4 and 5, Table 2**): follicular cadmium was detected in all six samples, iron in five samples, lead in four samples, and nickel in two samples. Some background silver was seen in two samples. Chromium, aluminium, bismuth and gold were not detected in any samples.

**Table 2:** Potentially toxic metals found by LA-ICP-MS in six human thyroid glands.

ID	Site	Hg	Cd	Pb	Fe	Ni	Ag	Cr	Al	Bi	Au
T40	Follicular	+	+	+	+	+	-	-	-	-	-
	Background	-	-	-	-	-	+	-	-	-	-
T86	Follicular	+	+	-	+	-	-	-	-	-	-
	Background	-	-	-	-	-	-	-	-	-	-
T91	Follicular	+	+	-	-	-	-	-	-	-	-
	Background	-	+	-	+	-	-	-	-	-	-
T97	Follicular	-	+	-	+	-	-	-	-	-	-
	Background	-	+	-	+	-	-	-	-	-	-
T45	Follicular	-	+	+	+	-	-	-	-	-	-
	Background	-	+	-	-	-	-	-	-	-	-
T70	Follicular	-	+	+	+	-	-	-	-	-	-
	Background	-	-	-	-	-	+	-	-	-	-

Follicular: follicular epithelial cells, ID: sample identification number,—not detected, + detected.

## Discussion

A key finding of this study is that mercury is commonly present in human adult thyroid follicular cells, raising the possibility that mercury could contribute to several thyroid disorders (**S1 Fig**). Other toxic metals such as cadmium and lead are also found in the human thyroid, suggesting synergistic interactions between toxic metals could enhance mercury toxicity in thyroid cells [24].

We were unable to ascertain why usually only some follicular cells contained mercury, but this variability in cellular mercury appears to be common in human tissues such as the brain [25], pituitary [26], pancreas [27] and breast [28]. Of note, follicular cells within a single follicle can be flattened on one side and cuboidal or columnar on the other, indicating the presence of functional polarity [23]. Theoretically, either increased mercury uptake or decreased elimination in subsets of follicular cells could underlie this variability. Future studies of autometallography combined with immunohistochemistry for a range of transporters of mercury both into and out of cells [29, 30] would be needed to see if variability in these cellular transporters underlies the patchy presence of mercury in follicular cells.

Toxic heavy metals such as mercury could trigger a variety of thyroid disorders since mercury can initiate pathways leading to genetic mutations [31, 32], autoimmune reactions [33], and oxygen free radical production [34, 35], mechanisms suspected to underlie the pathogenesis of thyroid cancers [36], autoimmune thyroiditis [37], and hypothyroidism [38]. Mercury has genotoxic properties that could promote the formation of cancer-causing mutations [31, 32]. Mouse thyroid stem cells contain the mercury transporter ABCG2/BCRP, which suggests thyroid stem cells (though not follicular cells that lack ABCG2/BCRP) have adapted to mercury uptake and make efforts to rid themselves of this metal [39, 40]. Mercury is a known promoter of autoimmune reactions [33, 41], so when the amount of mercury in thyroid follicular cells reaches a critical level in people with a genetic predisposition to autoimmunity, autoimmune thyroiditis could result [42, 43]. Some cases of hypothyroidism are thought to arise from subclinical autoimmune thyroiditis [38], and the generation of oxygen free radicals by mercury [35] is a further mechanism by which this metal could contribute to thyroid underactivity [44]. Evidence for a link between thyroid follicular cell mercury and oxidative stress could be sought by combining autometallography with a histochemical marker of nucleic acid oxidative damage [45]. A link between autoimmune thyroiditis and thyroid cancer has been noted by several researchers, with the proposal that the cancer may arise from the chronic inflammation of the thyroiditis [46, 47]; our finding of mercury in follicular cells suggests that mercury toxicity underlying both disorders could be another reason these two disorders often co-exist.

Increases over time in the incidence of thyroid cancer, autoimmune thyroiditis, and hypothyroidism have been reported [1, 13–17]. The amount of mercury in the global atmosphere has increased since 1950 [48], mostly due to the burning of coal [49]. Atmospheric mercury enters water and then seafood, especially in predatory fish where tissue mercury levels are rising [50]. Fish consumption is the commonest cause of human mercury exposure [51], and our finding that mercury is often present in thyroid cells suggests this metal is a candidate to explain the increasing incidence of a variety of thyroid disorders.

Of the non-mercury metals found in the thyroid, cadmium was most common, being present in the follicular cells of all LA-ICP-MS samples. Cadmium is a known genotoxin that has been implicated in thyroid cancer [52] and has endocrine-disrupting activities that could interfere with thyroid function [8]. Cigarette smoke is a major source of cadmium exposure in humans, but smoking appears to reduce rather than increase the risk of thyroid cancer [1], with contradictory evidence concerning its role as a risk factor for autoimmune thyroiditis [3, 42, 53] and hypothyroidism [3, 7, 53]. Although from our LA-ICP-MS images it appears likely that mercury and cadmium are co-located in some follicular cells, we cannot be sure this is the case since the spot size for LA-ICP-MS is 50 μm, which is larger than a typical cuboidal follicular cell (about 10 μm, see Fig 1F). Other metals detected in the thyroid on LA-ICP-MS were lead, iron, nickel and silver. Studies of the association of human thyroid hormones and lead exposure have given inconsistent results [7] and a meta-analysis has not provided evidence that occupational lead exposure affects thyroid function [54]. However, lead may recruit antibodies that attack the thyroid [55], and could play a part in autoimmune thyroiditis [56] and colloid goitre [57]. Iron is an essential metal but in large quantities, such as found in hemochromatosis, it may cause hypothyroidism with antithyroid antibodies [58]. A systemic allergy to nickel could result in an autoimmune reaction in people who have nickel in their thyroid cells [59]. Silver nanoparticles can induce oxidative stress in cells [60]. Finally, all these metals could interact with mercury to enhance thyroid cellular toxicity [24].

Human exposure to environmental toxic metals is common, so it is likely that other risk factors are needed to interact with metal toxicants before thyroid cells are damaged. Several metabolic processes act to protect cells from metal toxicants, and genetic variants affecting proteins in these pathways have been identified that could increase susceptibility to metals such as mercury [11]. Selenium detoxifies cellular mercury, and low selenium levels have been implicated in thyroid autoimmunity, cancer, and colloid goitre [56, 57, 61, 62]. However, we have no evidence from our study that follicular intracellular selenium levels were low, since LA-ICP-MS is not a sensitive method of detecting selenium and no histological methods are available to detect this element.

This study has several limitations. (**1**) Our forensic/coronial autopsy series provided thyroid samples from people over a wide range of ages and from varied clinic pathological conditions. However, no autopsy series can precisely replicate prevalence data from a living human population. (**2**) We did not have enough clinical information to know if any of the people in our study had clinical thyroid disease. Future prospective elemental studies of thyroid tissue from people with clinical data, thyroid function studies and thyroid autoantibodies would be needed to correlate individual thyroid disorders with metal content of the thyroid gland. (**3**) Prospective *in vivo* biochemical and genomic studies will be needed to examine the roles of selenium deficiency and genetic susceptibilities as susceptibility factors for metal toxicity in the thyroid. (**4**) Nobody in our study population had thyroid cancer. A future study looking at the heavy metal content of structurally normal thyroid tissue adjacent to thyroid cancers, as has been undertaken for breast cancer and mercury [28], will be needed to determine whether genotoxic metals are more commonly found in thyroid tissue from people with, compared to those without, thyroid cancer. (**5**) We were unable to determine why mercury enters thyroid follicular cells selectively. The presence of sulfhydryl groups and metallothionein in thyroid cells, which bind metals such as mercury and cadmium, could be one factor in the accumulation and persistence of these metals in thyroid cells. Future studies on toxic metals in the thyroid and their relationship to the thyroid content of sulfhydryl groups and to metallothionein expression could provide more information on this issue. (**6**) Autometallography can detect only inorganic mercury, but since this appears to be the proximate toxic form of mercury in cells [63] it is the most important form to identify.

In conclusion, mercury is found commonly in follicular cells of the human thyroid, the proportion of people having mercury in their thyroid follicular cells increases with aging, and other toxic metals such as cadmium are found often in the thyroid. Many toxic metals have damaging actions which may contribute to the pathogenesis of thyroid cancer, autoimmune thyroiditis, and hypothyroidism. Most effects of toxic metals on the human thyroid remain hypothetical (**S1 Fig**), so future prospective experiments correlating the presence of toxic metals in the thyroid with specific thyroid disorders will be needed to shed further light on the role toxic metals such as mercury play in thyroid diseases.

## Supporting information

S1 Fig Hypothetical pathway indicating how mercury and other toxic metals could increase the risk of thyroid disorders. Human exposure to mercury from (1) consumption of marine or freshwater fish, crustaceans and molluscs, (2) occupations, or (3) dental amalgam fillings results in inorganic or methylmercury being deposited in thyroid follicular cells. Methylmercury is slowly converted into inorganic mercury in cells. The toxicity of mercury could be enhanced by genetically susceptibilities, selenium deficiency, or the presence of other toxic metals. After bioaccumulation, critical intracellular level of mercury could produce genetic mutations triggering cancer, autoimmune reactions causing thyroiditis and hypothyroidism, and oxidative damage contributing further to hypothyroidism.
(TIF)

## References

[1] Kitahara CM, Sosa JA. The changing incidence of thyroid cancer. Nat Rev Endocrinol. 2016;12: 646–653. 10.1038/nrendo.2016.110 27418023 PMC10311569

[2] Hansen PS, Brix TH, Iachine I, Kyvik KO, Hegedus L. The relative importance of genetic and environmental effects for the early stages of thyroid autoimmunity: a study of healthy Danish twins. Eur J Endocrinol. 2006;154: 29–38. 10.1530/eje.1.02060 16381988

[3] Ferrari SM, Fallahi P, Antonelli A, Benvenga S. Environmental Issues in Thyroid Diseases. Front Endocrinol (Lausanne). 2017;8: 50 10.3389/fendo.2017.00050 28373861 PMC5357628

[4] Rana SV. Perspectives in endocrine toxicity of heavy metals—a review. Biol Trace Elem Res. 2014;160: 1–14. 10.1007/s12011-014-0023-7 24898714

[5] Malandrino P, Russo M, Ronchi A, Moretti F, Giani F, Vigneri P, et al Concentration of Metals and Trace Elements in the Normal Human and Rat Thyroid: Comparison with Muscle and Adipose Tissue and Volcanic Versus Control Areas. Thyroid. 2020;30: 290–299. 10.1089/thy.2019.0244 31880996

[6] Zhu X, Kusaka Y, Sato K, Zhang Q. The endocrine disruptive effects of mercury. Environ Health Prev Med. 2000;4: 174–183. 10.1007/BF02931255 21432482 PMC2723593

[7] Chen A, Kim SS, Chung E, Dietrich KN. Thyroid hormones in relation to lead, mercury, and cadmium exposure in the National Health and Nutrition Examination Survey, 2007–2008. Environ Health Perspect. 2013;121: 181–186. 10.1289/ehp.1205239 23164649 PMC3569681

[8] Buha A, Matovic V, Antonijevic B, Bulat Z, Curcic M, Renieri EA, et al Overview of Cadmium Thyroid Disrupting Effects and Mechanisms. Int J Mol Sci. 2018;19 10.3390/ijms19051501 29772829 PMC5983752

[9] Hansen JC, Reske-Nielsen E, Thorlacius-Ussing O, Rungby J, Danscher G. Distribution of dietary mercury in a dog. Quantitation and localization of total mercury in organs and central nervous system. Sci Total Environ. 1989;78: 23–43. 10.1016/0048-9697(89)90020-x 2717923

[10] Khayat A, Dencker L. Organ and cellular distribution of inhaled metallic mercury in the rat and Marmoset monkey (Callithrix jacchus): influence of ethyl alcohol pretreatment. Acta Pharmacol Toxicol (Copenh). 1984;55: 145–152. 10.1111/j.1600-0773.1984.tb01977.x 6437142

[11] Andreoli V, Sprovieri F. Genetic Aspects of Susceptibility to Mercury Toxicity: An Overview. Int J Environ Res Public Health. 2017;14: E93 10.3390/ijerph14010093 28106810 PMC5295343

[12] Joneidi Z, Mortazavi Y, Memari F, Roointan A, Chahardouli B, Rostami S. The impact of genetic variation on metabolism of heavy metals: Genetic predisposition? Biomed Pharmacother. 2019;113: 108642 10.1016/j.biopha.2019.108642 30849640

[13] Leese GP, Flynn RV, Jung RT, Macdonald TM, Murphy MJ, Morris AD. Increasing prevalence and incidence of thyroid disease in Tayside, Scotland: the Thyroid Epidemiology Audit and Research Study (TEARS). Clin Endocrinol (Oxf). 2008;68: 311–316. 10.1111/j.1365-2265.2007.03051.x 17970771

[14] Vanderpump MP. The epidemiology of thyroid disease. Br Med Bull. 2011;99: 39–51. 10.1093/bmb/ldr030 21893493

[15] McLeod DS, Cooper DS. The incidence and prevalence of thyroid autoimmunity. Endocrine. 2012;42: 252–265. 10.1007/s12020-012-9703-2 22644837

[16] Vigneri R, Malandrino P, Russo M. Is Thyroid Cancer Increasing in Incidence and Aggressiveness? J Clin Endocrinol Metab. 2020;105 10.1210/clinem/dgaa223 32369832

[17] Kim J, Gosnell JE, Roman SA. Geographic influences in the global rise of thyroid cancer. Nat Rev Endocrinol. 2020;16: 17–29. 10.1038/s41574-019-0263-x 31616074

[18] Guzzi G, Grandi M, Cattaneo C, Calza S, Minoia C, Ronchi A, et al Dental amalgam and mercury levels in autopsy tissues: food for thought. Am J Forensic Med Pathol. 2006;27: 42–45. 10.1097/01.paf.0000201177.62921.c8 16501347

[19] Bjorkman L, Lundekvam BF, Laegreid T, Bertelsen BI, Morild I, Lilleng P, et al Mercury in human brain, blood, muscle and toenails in relation to exposure: an autopsy study. Environ Health. 2007;6: 30 10.1186/1476-069X-6-30 17931423 PMC2098763

[20] Danscher G, Moller-Madsen B. Silver amplification of mercury sulfide and selenide: a histochemical method for light and electron microscopic localization of mercury in tissue. J Histochem Cytochem. 1985;33: 219–228. 10.1177/33.3.2579122 2579122

[21] Danscher G, Rungby J. Differentiation of histochemically visualized mercury and silver. Histochem J. 1986;18: 109–114. 10.1007/BF01675364 3733462

[22] Pamphlett R, Png FY. Shrinkage of motor axons following systemic exposure to inorganic mercury. J Neuropathol Exp Neurol. 1998;57: 360–366. 10.1097/00005072-199804000-00009 9600230

[23] Carcangiu ML (1997) Thyroid In: Sternberg SS, editor. Histology for Pathologists. 2nd ed. Philadelphia: Lippincott-Raven pp. 1075–1092.

[24] Andrade VM, Aschner M, Marreilha Dos Santos AP (2017) Neurotoxicity of Metal Mixtures In: Aschner M, Costa LG, editors. Neurotoxicity of Metals. 2017/09/11 ed. Cham, Switzerland: Springer Nature pp. 227–265. 10.1007/978-3-319-60189-2_12 28889271

[25] Pamphlett R, Mak R, Lee J, Buckland ME, Harding AJ, Kum Jew S, et al Concentrations of toxic metals and essential trace elements vary among individual neurons in the human locus ceruleus. PLoS One. 2020;15: e0233300 10.1371/journal.pone.0233300 32428015 PMC7237016

[26] Pamphlett R, Kum Jew S, Doble PA, Bishop DP. Elemental Analysis of Aging Human Pituitary Glands Implicates Mercury as a Contributor to the Somatopause. Front Endocrinol (Lausanne). 2019;10: 419 10.3389/fendo.2019.00419 31297094 PMC6607410

[27] Pamphlett R, Colebatch AJ, Doble PA, Bishop DP. Mercury in Pancreatic Cells of People with and without Pancreatic Cancer. Int J Environ Res Public Health. 2020;17 10.3390/ijerph17238990 33276658 PMC7731371

[28] Pamphlett R, Satgunaseelan L, Kum Jew S, Doble PA, Bishop DP. Elemental bioimaging shows mercury and other toxic metals in normal breast tissue and in breast cancers. PLoS One. 2020;15: e0228226 10.1371/journal.pone.0228226 32004334 PMC6993973

[29] Bridges CC, Zalups RK. Mechanisms involved in the transport of mercuric ions in target tissues. Arch Toxicol. 2017;91: 63–81. 10.1007/s00204-016-1803-y 27422290 PMC5226910

[30] Maliepaard M, Scheffer GL, Faneyte IF, van Gastelen MA, Pijnenborg AC, Schinkel AH, et al Subcellular localization and distribution of the breast cancer resistance protein transporter in normal human tissues. Cancer Res. 2001;61: 3458–3464. 11309308

[31] Crespo-Lopez ME, Macedo GL, Pereira SI, Arrifano GP, Picanco-Diniz DL, do Nascimento JL, et al Mercury and human genotoxicity: critical considerations and possible molecular mechanisms. Pharmacol Res. 2009;60: 212–220. 10.1016/j.phrs.2009.02.011 19446469

[32] Nersesyan A, Kundi M, Waldherr M, Setayesh T, Misik M, Wultsch G, et al Results of micronucleus assays with individuals who are occupationally and environmentally exposed to mercury, lead and cadmium. Mutat Res. 2016;770: 119–139. 10.1016/j.mrrev.2016.04.002 27894681

[33] Pollard KM, Cauvi DM, Toomey CB, Hultman P, Kono DH. Mercury-induced inflammation and autoimmunity. Biochim Biophys Acta Gen Subj. 2019;1863: 129299 10.1016/j.bbagen.2019.02.001 30742953 PMC6689266

[34] Pollard KM, Hultman P, Kono DH. Toxicology of autoimmune diseases. Chem Res Toxicol. 2010;23: 455–466. 10.1021/tx9003787 20078109 PMC3076021

[35] Tchounwou PB, Yedjou CG, Patlolla AK, Sutton DJ. Heavy metal toxicity and the environment. Exp Suppl. 2012;101: 133–164. 10.1007/978-3-7643-8340-4_6 22945569 PMC4144270

[36] Vigneri R, Malandrino P, Vigneri P. The changing epidemiology of thyroid cancer: why is incidence increasing? Curr Opin Oncol. 2015;27: 1–7. 10.1097/CCO.0000000000000148 25310641

[37] Perga S, Martire S, Montarolo F, Giordani I, Spadaro M, Bono G, et al The Footprints of Poly-Autoimmunity: Evidence for Common Biological Factors Involved in Multiple Sclerosis and Hashimoto’s Thyroiditis. Front Immunol. 2018;9: 311 10.3389/fimmu.2018.00311 29527211 PMC5829620

[38] Chaker L, Bianco AC, Jonklaas J, Peeters RP. Hypothyroidism. Lancet. 2017;390: 1550–1562. 10.1016/S0140-6736(17)30703-1 28336049 PMC6619426

[39] Hoshi N, Kusakabe T, Taylor BJ, Kimura S. Side population cells in the mouse thyroid exhibit stem/progenitor cell-like characteristics. Endocrinology. 2007;148: 4251–4258. 10.1210/en.2006-0490 17584961 PMC2582754

[40] Mato E, Gonzalez C, Moral A, Perez JI, Bell O, Lerma E, et al ABCG2/BCRP gene expression is related to epithelial-mesenchymal transition inducer genes in a papillary thyroid carcinoma cell line (TPC-1). J Mol Endocrinol. 2014;52: 289–300. 10.1530/JME-14-0051 24643400

[41] Maqbool F, Niaz K, Hassan FI, Khan F, Abdollahi M. Immunotoxicity of mercury: Pathological and toxicological effects. J Environ Sci Health C Environ Carcinog Ecotoxicol Rev. 2017;35: 29–46. 10.1080/10590501.2016.1278299 28055311

[42] Saranac L, Zivanovic S, Bjelakovic B, Stamenkovic H, Novak M, Kamenov B. Why is the thyroid so prone to autoimmune disease? Horm Res Paediatr. 2011;75: 157–165. 10.1159/000324442 21346360

[43] Duntas LH. Environmental factors and autoimmune thyroiditis. Nat Clin Pract Endocrinol Metab. 2008;4: 454–460. 10.1038/ncpendmet0896 18607401

[44] Mancini A, Di Segni C, Raimondo S, Olivieri G, Silvestrini A, Meucci E, et al Thyroid Hormones, Oxidative Stress, and Inflammation. Mediators Inflamm. 2016;2016: 6757154 10.1155/2016/6757154 27051079 PMC4802023

[45] Pamphlett R, Slater M, Thomas S. Oxidative damage to nucleic acids in motor neurons containing mercury. J Neurol Sci. 1998;159: 121–126. 10.1016/s0022-510x(98)00161-0 9741394

[46] Noureldine SI, Tufano RP. Association of Hashimoto’s thyroiditis and thyroid cancer. Curr Opin Oncol. 2015;27: 21–25. 10.1097/CCO.0000000000000150 25390557

[47] Resende de Paiva C, Gronhoj C, Feldt-Rasmussen U, von Buchwald C. Association between Hashimoto’s Thyroiditis and Thyroid Cancer in 64,628 Patients. Front Oncol. 2017;7: 53 10.3389/fonc.2017.00053 28443243 PMC5385456

[48] Streets DG, Devane MK, Lu Z, Bond TC, Sunderland EM, Jacob DJ. All-time releases of mercury to the atmosphere from human activities. Environ Sci Technol. 2011;45: 10485–10491. 10.1021/es202765m 22070723 PMC3246392

[49] Streets DG, Lu Z, Levin L, Ter Schure AFH, Sunderland EM. Historical releases of mercury to air, land, and water from coal combustion. Sci Total Environ. 2018;615: 131–140. 10.1016/j.scitotenv.2017.09.207 28964988

[50] Lavoie RA, Bouffard A, Maranger R, Amyot M. Mercury transport and human exposure from global marine fisheries. Sci Rep. 2018;8: 6705 10.1038/s41598-018-24938-3 29712952 PMC5928114

[51] Karimi R, Silbernagel S, Fisher NS, Meliker JR. Elevated blood Hg at recommended seafood consumption rates in adult seafood consumers. Int J Hyg Environ Health. 2014;217: 758–764. 10.1016/j.ijheh.2014.03.007 24780236

[52] Marotta V, Malandrino P, Russo M, Panariello I, Ionna F, Chiofalo MG, et al Fathoming the link between anthropogenic chemical contamination and thyroid cancer. Crit Rev Oncol Hematol. 2020;150: 102950 10.1016/j.critrevonc.2020.102950 32339980

[53] Kim SJ, Kim MJ, Yoon SG, Myong JP, Yu HW, Chai YJ, et al Impact of smoking on thyroid gland: dose-related effect of urinary cotinine levels on thyroid function and thyroid autoimmunity. Sci Rep. 2019;9: 4213 10.1038/s41598-019-40708-1 30862792 PMC6414657

[54] Krieg EF Jr. A meta-analysis of studies investigating the effects of occupational lead exposure on thyroid hormones. Am J Ind Med. 2016;59: 583–590. 10.1002/ajim.22591 27094769 PMC4934017

[55] Nie X, Chen Y, Chen Y, Chen C, Han B, Li Q, et al Lead and cadmium exposure, higher thyroid antibodies and thyroid dysfunction in Chinese women. Environ Pollut. 2017;230: 320–328. 10.1016/j.envpol.2017.06.052 28667913

[56] Stojsavljevic A, Rovcanin B, Jagodic J, Radojkovic DD, Paunovic I, Gavrovic-Jankulovic M, et al Significance of arsenic and lead in Hashimoto’s thyroiditis demonstrated on thyroid tissue, blood, and urine samples. Environ Res. 2020;186: 109538 10.1016/j.envres.2020.109538 32334172

[57] Stojsavljevic A, Rovcanin B, Jagodic J, Borkovic‑Mitic S, Paunovic I, Diklic A, et al Risk Assessment of Toxic and Essential Trace Metals on the Thyroid Health at the Tissue Level: The Significance of Lead and Selenium for Colloid Goiter Disease. Expos Health. 2020;12: 255–264. 10.1007/s12403-019-00309-9

[58] Edwards CQ, Kelly TM, Ellwein G, Kushner JP. Thyroid disease in hemochromatosis. Increased incidence in homozygous men. Arch Intern Med. 1983;143: 1890–1893. 6625774

[59] Andrioli M, Trimboli P, Maio D, Persani L, Minelli M. Systemic nickel allergic syndrome as an immune-mediated disease with an increased risk for thyroid autoimmunity. Endocrine. 2015;50: 807–810. 10.1007/s12020-015-0581-2 25795291

[60] Mao BH, Chen ZY, Wang YJ, Yan SJ. Silver nanoparticles have lethal and sublethal adverse effects on development and longevity by inducing ROS-mediated stress responses. Sci Rep. 2018;8: 2445 10.1038/s41598-018-20728-z 29402973 PMC5799281

[61] Duntas LH. The role of selenium in thyroid autoimmunity and cancer. Thyroid. 2006;16: 455–460. 10.1089/thy.2006.16.455 16756467

[62] Ventura M, Melo M, Carrilho F. Selenium and Thyroid Disease: From Pathophysiology to Treatment. Int J Endocrinol. 2017;2017: 1297658 10.1155/2017/1297658 28255299 PMC5307254

[63] Clarkson TW, Magos L. The toxicology of mercury and its chemical compounds. Crit Rev Toxicol. 2006;36: 609–662. 10.1080/10408440600845619 16973445

