# Supplementary figures and images for: Mercury in the human thyroid gland: Potential implications for thyroid cancer, autoimmune thyroiditis, and hypothyroidism
Source: PLoS One. 2021 Feb 9;16(2):e0246748. doi: 10.1371/journal.pone.0246748 (PMC7872292; doi:10.1371/journal.pone.0246748)

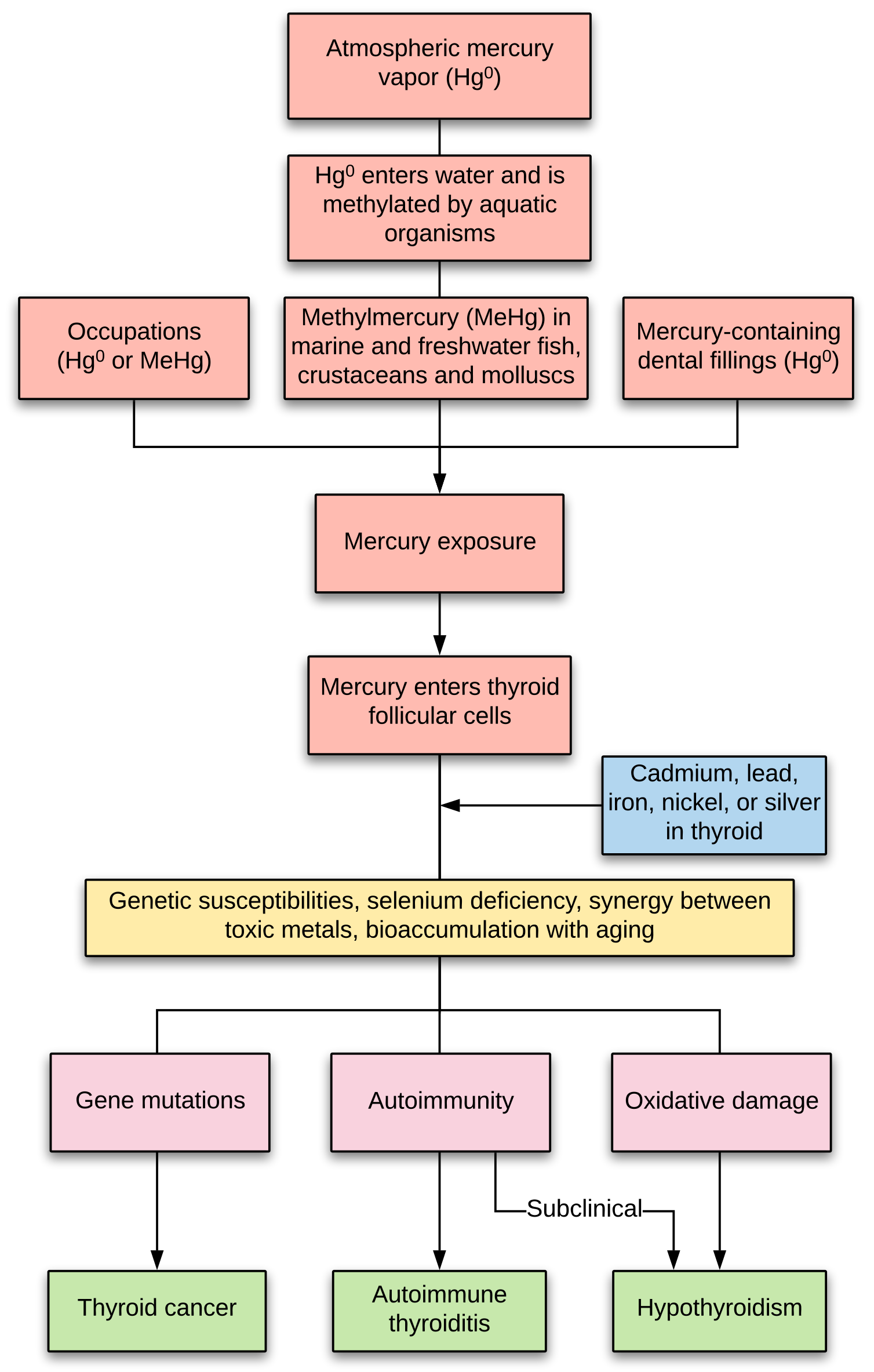

Supplement: S1 Fig — Human exposure to mercury from (1) consumption of marine or freshwater fish, crustaceans and molluscs, (2) occupations, or (3) dental amalgam fillings results in inorganic or methylmercury being deposited in thyroid follicular cells. Methylmercury is slowly converted into inorganic mercury in cells. The toxicity of mercury could be enhanced by genetically susceptibilities, selenium deficiency, or the presence of other toxic metals. After bioaccumulation, critical intracellular level of mercury could produce genetic mutations triggering cancer, autoimmune reactions causing thyroiditis and hypothyroidism, and oxidative damage contributing further to hypothyroidism. (TIF) [file pone.0246748.s001.tif]
